# Ce^3+^, Pr^3+^ Co-Doped Lu_3_Al_5_O_12_ Single Crystals and Ceramics: A Comparative Study

**DOI:** 10.3390/ma15249025

**Published:** 2022-12-17

**Authors:** Yifei Xiong, Yun Shi, Haibo Wang, Qian Zhang, Tong Wu, Qiang Yuan, Kaicheng Ma, Tongtong Li, Zhenzhen Zhou, Jinghong Fang, Huan He, Jinqi Ni, Qian Liu, Jiangding Yu, Sheng Cui, Oleg Shichalin, Eugeniy Papynov

**Affiliations:** 1 School of Material Science and Engineering, Nanjing Tech University, Nanjing 211816, China; 2 State Key Laboratory of High Performance Ceramics and Superfine Microstructure, Shanghai Institute of Ceramics, Chinese Academy of Sciences, Shanghai 200050, China; 3 Center of Materials Science and Optoelectronics Engineering, University of Chinese Academy of Sciences, Beijing 100049, China; 4 Department of Nuclear Technology, Institute of Science-Intensive Technologies and Advanced Materials, Far Eastern Federal University, 10 Ajax Bay, Russky Island, Vladivostok 690922, Russia

**Keywords:** Ce, Pr:LuAG, optical floating zone method, scintillator ceramics, garnet, single crystals

## Abstract

Ce^3+^, Pr^3+^ co-doped Lu_3_Al_5_O_12_ (Ce, Pr:LuAG) single crystals and ceramics were prepared using the optical floating zone (OFZ) and reactive vacuum sintering methods, respectively. The microstructure, photo- (λ_ex_ = 450 nm), and radio-luminescence (under X-ray excitation) performance, as well as scintillation light yield (LY, under γ-ray, ^137^Cs source) of both materials, were investigated and compared. Ce, Pr:LuAG ceramics had an in-line transmittance of approximately 20% in the visible light range, while the analogous crystals were more transparent (~65%). The X-ray excited luminescent (XEL) spectra showed the characteristic Ce ^3+^ and Pr^3+^ emissions located at 310 nm, 380 nm, and 510 nm. The highest LY of the Ce, Pr:LuAG ceramics reached 34,112 pho/MeV at 2 μs time gate, which is higher than that of a single crystal. The ratio of LY values (LY2/LY0.75) between shaping times of 0.75 μs and 2 μs indicated a faster scintillation decay of ceramics regarding single crystals. It was ascribed to the lower effective concentration of luminescent activators in single crystals because of the coefficient segregation effect.

## 1. Introduction

Scintillator materials can convert incoming high-energy rays or particles into visible or ultraviolet light [1]. They have been widely used as an energy transfer in nuclear medicine imaging, high energy physics, industrial nondestructive inspection, well logging, as well as homeland security fields. The new generation of scintillators are desired to have high density, fast decay, and high scintillation efficiency. Lu_3_Al_5_O_12_ (LuAG) belongs to the garnet crystal system having a body-centered cubic lattice structure; its density (6.67 g/cm^3^) and effective atomic number (Z_eff_ = 63) are high, which means an effective ability to stop or absorb high energy rays or particles. LuAG thus, is considered to be a desirable host for functional regulation as scintillators for the application of X-ray or γ ray detection [2,3].

Ce^3+^ and Pr^3+^, having 4f–5d configuration, are the activators with high luminescence efficiency and fast decay. Ce^3+^ or Pr^3+^ doped LuAG crystals [3,4], ceramics [5,6], fibers [7], and films [8] have been extensively investigated. Based on the Bartram−Lempicki model, the theoretical LY reached 60,000 pho/MeV [9,10]. The emission of Ce^3+^ doped LuAG locating at 540 nm, matches well with the silicon photodiode (Si-PD), and its decay is fast (~60 ns). Furthermore, Ce:LuAG is considered a potential candidate for scintillators for future high-energy physics experiments due to its hard radiation tolerance [11,12]. Pr:LuAG has the main emission at 310 nm and very fast decay (~20 ns); it is considered one of the high-quality scintillators for the application of TOF-PET (Time of Flight, Positron Emission Tomography) [6,13]. Meanwhile, due to the high luminescence thermal stability of Pr:LuAG [14], its potential in the well logging field has also been widely concerned [15].

Considering that in the LuAG host, the 310 nm emission of Pr^3+^ falls right in the absorption band of Ce^3+^, the possible energy transfer between Pr^3+^ and Ce^3+^ has attracted research interests. Zhou et al. [16] investigated the Ce^3+^ and Pr^3+^ co-doped LuAG transparent ceramics by setting Pr^3+^ concentration at 0.25 at.% while changing Ce^3+^ concentration from 0 to 0.3 at.%. The co-doping of Pr^3+^ in Ce:LuAG was found to be able to enhance the Ce^3+^ emission intensity of 550 nm, which reached a maximum in 0.2 at.% Ce, and 0.25 at.% Pr co-doped LuAG. They ascribed it to the energy transfer from Pr^3+^ to Ce^3+^. They also grew Ce, Pr:LuYAG crystals [17] and pointed to their potential application in white LED lighting. More interestingly, because the emission wavelengths of the Pr^3+^ and Ce^3+^ ions are well separated, Ce, Pr:LuAG was recently used as a high-precision temperature imaging technique [18,19,20]. However, previous studies have shown that the antisite defect [3] in LuAG would introduce slow scintillation decay components and decrease scintillation efficiency in both Ce: LuAG and Pr:LuAG crystals. Liu et al. [21] proposed an Mg^2+^ co-doping strategy, which successfully shortened the decay time while at the expense of scintillation light yield since the Mg^2+^ ion would not contribute to Ce^3+^ emission. The decay of Pr^3+^ in LuAG is about 20 ns which is faster than that of Ce:LuAG (~ 60 ns). Furthermore, considering that the energy transfer existed between Ce^3+^ and Pr^3+^, it would be potential to improve the scintillation performance of LuAG by the co-doping of Ce^3+^ and Pr^3+^, whereas there were few reports of such work except the ref. [16,17] till now.

Compared with single crystals grown by the traditional method, such as Czochralski (CZ) method [22] or Bridgman method [23], etc., transparent ceramics [5] were proposed to have comparable optical quality and be able to save both time and raw materials, especially when doing composition exploration. However, the OFZ method, as a containerless technology for fast crystal growth, has attracted research interests in recent years. A series of crystal scintillators, such as Ce^3+^ doped Gd_3_(Ga, Al)_5_O_12_ (Ce: GGAG) [23], SrHfO_3_ (SHO), and SrZrO_3_ (SZO) [24], Ce: (La, Gd)_2_Si_2_O_7_ crystals [25], etc., have been grown by OFZ method successively.

In this work, we investigated the growth of Ce, Pr co-doped LuAG crystals by OFZ method, which was rarely involved in previous works, and a comparative study with Ce, Pr:LuAG ceramics was also conducted by investigating their microstructure, optical quality, photo-and radio- luminescence (PL and XEL) performance, as well as their scintillation LY and decay under γ- ray excitation.

## 2. Materials and Methods

### 2.1. Materials Preparation

The commercial oxide powders of Diyang Corp. (Shanghai, China) with high purity (>99.99%), CeO_2_, Pr_6_O_11_, Lu_2_O_3_, and Al_2_O_3_, were used as raw materials; they were weighted according to the chemical stoichiometric of (Ce_0.005_Pr_0.001_Lu_0.094_)_3_Al_5_O_12_ (0.5 at.% Ce, 0.1 at.% Pr:LuAG). The mixed powders were wet milled in a planetary ball mill apparatus using alumina mill pots and balls. We used high pure alcohol as ball mill media. After 12 h of ball milling at a 200 rpm rate, the slurry was processed by the following 70 °C -dry and 200-mesh sieving. The powder products were then calcined at 600 °C for 4 h in air to eliminate the possibly introduced organic impurities. They were then used for ceramics fabrication and crystal growth of Ce, Pr:LuAG, respectively.

The ceramics were fabricated by a solid-state reaction method through vacuum sintering in a tungsten crucible. The detailed process was described in our previous works [5,26]. In addition, to keep composition conditions between ceramics and crystals as similar as possible, we did not use any sintering aids in this work. Furthermore, to eliminate the oxygen vacancies which were possibly formed during the vacuum sintering, the as sintered ceramics were annealed in air at 1450 °C for 10 h in a MoSi_2_ furnace. The temperature and holding time were previously used as a proper annealing conditions in Ce:LuAG and Pr:LuAG ceramics [5,27].

For the crystal growth, the powders were molded to rods with a dimension of Ø 8 mm × 100 mm for cold isostatic pressing treatment under 70 MPa for 20 min. Then, the ceramic rods were sintered in the tube furnace at 1600 °C for 8 h with a flowing Ar + 5% H_2_. The crystal growth was conducted successively in an optical floating zone furnace (FZ-T1000 H CSC Cop., Tokyo, Japan) equipped with four halogen lamps (1000 W) as a heating source, crucible free. The crystal growth atmosphere was Ar + 5% H_2_; the growth rate was 3–5 mm/h, and the rotation rate was 20 rpm. The seed crystal was a commercial Y_3_Al_5_O_12_ (YAG) crystal rod with <111> orientation, and the ceramic rods were used as feed rods. The whole crystal growth process was monitored by an in situ image system. Considering the cracks and possibly existing inner stresses introduced by the high-temperature gradient of the OFZ method, the annealing effect was also investigated. Moreover, to avoid the possible propagation of cracks because of the inner stress in crystals, we take the low-temperature annealing condition of 1000 °C in air for 5 h and 10 h in a MoSi_2_ furnace, 1 °C/min heating and cooling rate, respectively, which was normally used as a crystal annealing condition.

The as sintered Ce, Pr:LuAG ceramics were cut and double-face polished to 4 × 4 × 1.2 mm^3^, and the as grown Ce, Pr:LuAG crystals were also cut and double-face polished to 2 mm dimension for measurement.

### 2.2. Characterization

The X-ray diffraction (XRD) patterns of the Ce, Pr:LuAG crystals and ceramics were indexed by the Rigaku Ultima IV diffractometer (Cu Kα, 40 kV, 40 mA, Rigaku Ultima IV, Japan) in the 2θ range of 20–90°, the scanning speed was 5°/min. The crystal quality was evaluated by a self-assembling Laue camera instrument (X-ray tube 2 kW, 60 kV). The microstructure and elements distribution of ceramics were measured by a high-resolution scanning electron microscope (SEM) equipped with an energy-dispersive X-ray spectroscope (EDS) (S-3400 N, Hitachi, Tokyo, Japan). The fine elements changing between the nominal chemical stochiometric and the final as-sintered ceramics or as-grown crystals were characterized by high-resolution inductively coupled plasma atomic emission spectrometry (ICP-OES). The absorbance and in-line transmittance spectra of the crystals and ceramics were measured using a UV–vis−NIR photometer (Varian Cary 5000), and the apparatus baseline was subtracted automatically. Photoluminescence (PL) and excitation (PLE) spectra were characterized by a Hitachi F-4600 fluorescence spectrometer. Steady-state radioluminescence spectra of the samples were characterized by the self-assembled X-ray fluorescence spectrometer (X-ray tube: 70 kV, 1.5 mA). Pulse height spectra under γ ray excitation (662 keV, ^137^Cs source) were detected by a Hamamatsu R878 photomultiplier (1 kV) using 0.75 μs, 1 μs, 2 μs shaping time, respectively. All the samples were directly tested at room temperature.

## 3. Results and Discussions

### 3.1. Analysis of Crystal Quality

**Figure 1:**
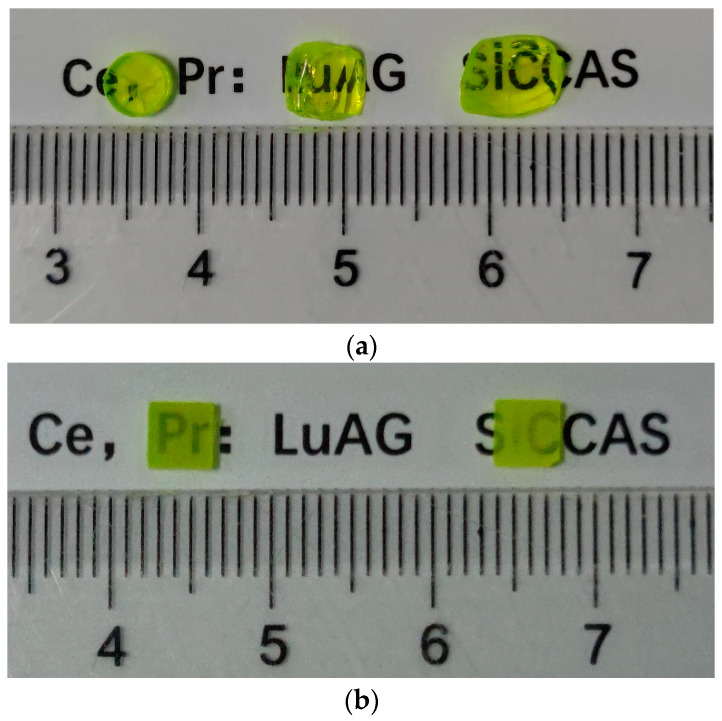
(**a**) Photograph of the Ce, Pr:LuAG crystals, as grown, with air annealing 1000 °C—5 h, 1000 °C—10 h from left to right, respectively (2.0 mm thickness) and (**b**) unannealed (left) and annealed (right) Ce, Pr:LuAG ceramics (1.2 mm thickness).

Figure 1 gives the photograph of the Ce, Pr:LuAG crystals and ceramics after being double-face polished. It shows that the Ce, Pr:LuAG crystals and ceramics are all green yellow in color, and after the air annealing process, they are brighter. In Figure 1a, the three crystals pieces (from left to right) were cut in three different positions along the growth direction (from bottom to up). Cracks can be observed in the as grown Ce, Pr:LuAG crystals which were introduced by the high-temperature gradient of the OFZ method. The as sintered Ce, Pr:LuAG ceramics are translucent, see Figure 1b, which could attribute to the lack of sintering aids (S.A.) in those ceramics since the S.A. would promote the densification process during vacuum sintering and thus help to eliminate the micropores sufficiently. The micropores were usually considered as the main light sources in ceramics, which would decrease the transparency [2,5].

**Figure 2:**
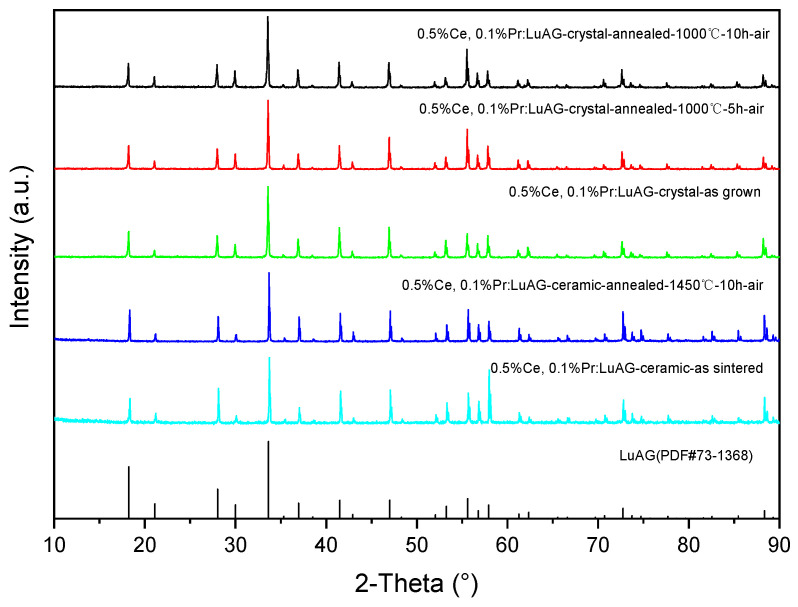
XRD patterns of the as grown Ce, Pr:LuAG crystal powders and bulk ceramic.

**Figure 3:**
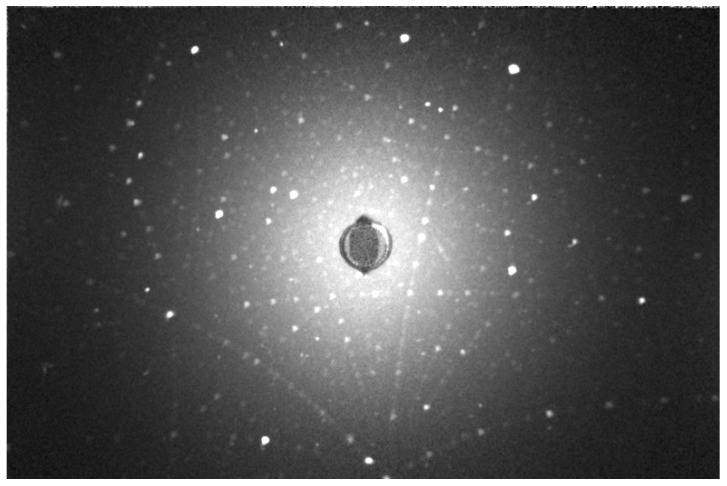
The Laure photograph of the as grown Ce, Pr:LuAG crystals.

The Ce, Pr:LuAG crystals were ground to powders for the XRD test. Their powder XRD patterns are shown in Figure 2. Both the patterns of crystal powders and bulk ceramics were well indexed by the cubic LuAG phase (PDF: No. 73-1368), and no detectable second phase can be observed. The high-temperature air annealing process of ceramics (1450 °C—10 h—air) has no effect on second-phase formation. Laue photograph in Figure 3 reveals that the as grown Ce, Pr:LuAG are single crystals with a high degree of crystallization, although there are cracks at the macro level.

**Figure 4:**
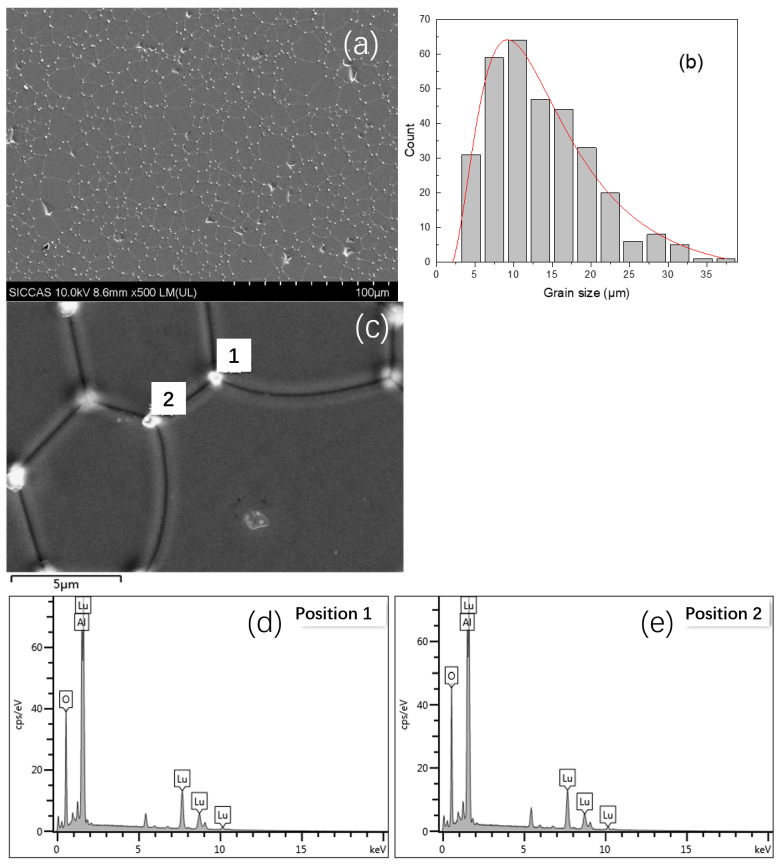
(**a**) SEM graph of Ce, Pr:LuAG ceramic after thermal etched at 1500 °C for 3 h in air with a slightly flowing O_2_; (**b**) grain size distribution; (**c**–**e**) element analysis of position 1 and position 2 of the triple grain boundary junctions by EDS, respectively.

Figure 4a gives the microstructure of Ce, Pr:LuAG ceramic after thermal etched at 1500 °C for 3 h in air with slightly flowing O_2_. Micro-pores can be observed, which means that the microstructure is not sufficiently dense and thus leads to the low transparency of those ceramics. By the Linear intercept method, the average grain size was calculated to be 13.6 μm. The grain size distribution of the Ce, Pr:LuAG ceramic was also calculated by 319 grains, and shown in Figure 4b, the grain size was mainly distributed at approximately 10 μm. It is interesting to note that shining spots existed in the triple grain boundary junctions, which was rarely reported before. The EDS investigation reveals that they are composition segregation of Lu_2_O_3_ and Al_2_O_3_, see Figure 4c–e. The traces of impurities revealed by the EDS elements analysis means the existence of local non-stoichiometric region or second phase, but the content should be too low to be detected by XRD technology since the detection limit of which is around 1%. The unmarked peaks in Figure 4d,e should be ascribed to a plated conductive Cr film on the surface of the ceramic samples, so they were not labeled.

Table 1 gives the experimental weight concentration (wt.%) of Ce^3+^ and Pr^3+^ in the obtained ceramics and crystals by ICP-OES measurement. The nominal concentration of Ce^3+^ and Pr^3+^ in this work is 0.5 at.% and 0.1 at.%, which can be calculated to be 0.25 wt.% and 0.05 wt.%, respectively. As shown in Table 1, the as sintered ceramics kept the Ce^3+^ and Pr^3+^ concentrations well with nominal content, while for crystals, the real concentrations are much lower than that of nominal concentrations. In addition, the real concentrations at different positions are different. Significantly, the lower activator concentration in the annealed crystals is not because of the post-air annealing process but should be ascribed to the uniformity of the crystals in different positions caused by the coefficient segregation effect of single crystals [28].

### 3.2. Optical and Luminescence Performance

**Figure 5:**
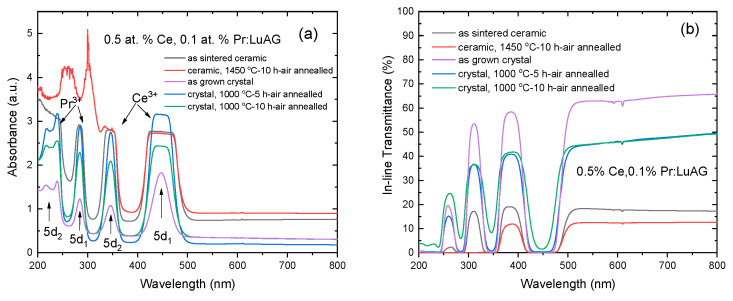
Absorbance spectra (**a**) and in line transmittance (**b**) of the as grown Ce, Pr:LuAG crystals (2 mm thickness) and ceramics (1.2 mm thickness).

**Figure 6:**
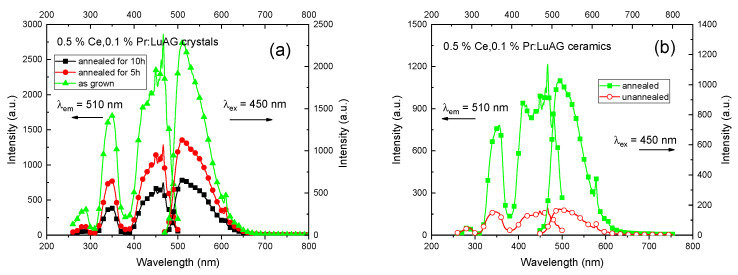
PL and PLE spectra of the (**a**) Ce, Pr:LuAG crystals and (**b**) Ce, Pr:LuAG ceramics with and without the post annealing process. λ_em_ = 510 nm, λ_ex_ = 450 nm.

Figure 5 shows the absorbance and in-line transmittance spectra of the Ce, Pr:LuAG crystals and ceramics. The absorption bands from Ce^3+^ and Pr^3+^ were found to co-exist and were mainly located in the range of 200–500 nm. The two strong absorption peaks at 240 nm and 285 nm correspond to the transition from the 4f_2_(^3^H_4_) level to the 5d_1,2_ level of Pr^3+^ ions [6], while the two absorption peaks at 346 and 450 nm are the characteristic absorption of Ce^3+^, which are caused by the transition from ^2^F_5/2_ of the 4f ground state of Ce^3+^ to the 5d_1_ and 5d_2_ levels [29], respectively. As shown in Figure 6b, the in-line transmittance of the as grown Ce, Pr:LuAG crystal reaches approximately 65% (2 mm thickness, double face polished) at visible light range. We suspect that the cracks (see Figure 1) and possible inclusions inside the crystals might be the possible light scattering sources. The transmittance difference between the annealed and unannealed crystals can be attributed to the uniformity between the different positions of the crystal rods. In future work, the optical quality of the crystals needs to be optimized by investigating the growth parameter.

The absorbance spectra of the as sintered Ce, Pr:LuAG ceramics and crystals revealed the characterized peaks, which are related to the 4f–5d transition of Ce^3+^ and Pr^3+^ ions. However, after the air annealing process, additional absorption at 257 nm and 300 nm can be observed in ceramics. The absorption peak at 257 nm might be ascribed to the presence of Ce^4+^ charge transfer (CT) [5]. The 300 nm was never reported [30], and its mechanism needs to be confirmed. The in-line transmittance in the visible light range of Ce, Pr:LuAG ceramics is low (~10–20%), which is related to the absence of sintering aids during the vacuum sintering, see Figure 5b. The high optical transmittance, particularly in the visible light region, is highly desirable as it allows for a greater amount of emitted light to reach the photodetector detector, thus enhancing the detection efficiency.

Figure 6 gives the PLE (λ_em_ = 510 nm) and PL (λ_ex_ = 450 nm) spectra of the Ce, Pr:LuAG crystals and ceramics. The shape and position of their PL and PLE bands are similar, which demonstrated a characteristic emission of Ce^3+^ and Pr^3+^, respectively. PL intensities of crystals with air annealing process is lower than that of as grown crystal. It is reasonable since the real concentration of Ce^3+^ or Pr^3+^ in the obtained crystals with air annealing process is lower than that of as grown crystals due to the segregation coefficient effect caused by uniformity which has been elucidated by ICP-OES, see Table 1. While in ceramics, as expected, the air annealing enhanced the PL intensity. It can be attributed to the successful removal of oxygen vacations or color centers in ceramics [31].

**Figure 7:**
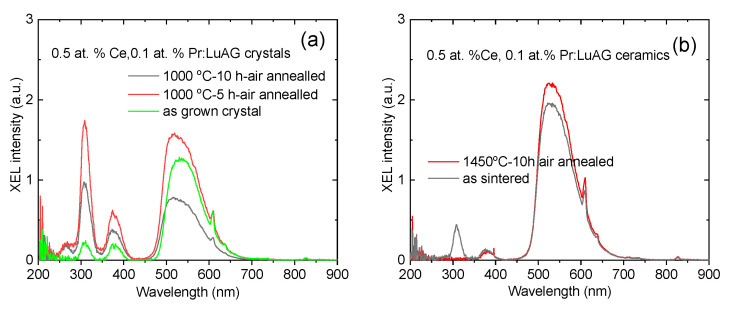
XEL spectra of the as grown Ce, Pr:LuAG crystals (**a**) and ceramics (**b**).

The RL luminescence spectra under X-ray excitation were revealed in Figure 7. Characteristic Ce^3+^ emission [5] and Pr^3+^ emission [6] were found to co-exist and well separate, although the emission pattern in the 200 nm–400 nm range of crystal and ceramic is different. In crystals, the emission at 380 nm was enhanced to be comparable with 310 nm, which is different from Pr:LuAG [6]. In ceramics, the emission peaking at 550 nm, which is mainly ascribed to the 5d–4f transition of Ce^3+^, is much stronger than the emissions of Pr^3+^, located mainly at around 310 nm and 380 nm. The absence of 310 nm emission of the annealed Ce, Pr:LuAG ceramics can be ascribed to the self-absorption at 300 nm, as shown in Figure 6a.

### 3.3. Scintillation Performance

**Figure 8:**
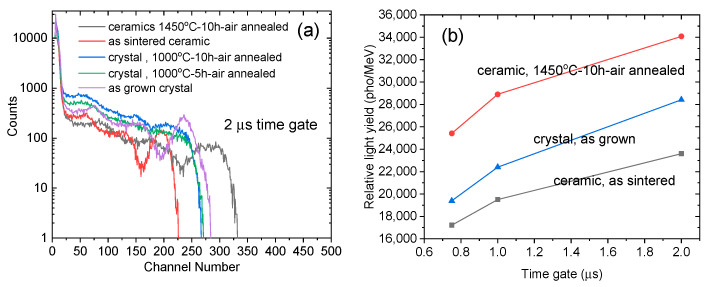
(**a**) Pulse height spectra of the as grown Ce, Pr:LuAG crystals and ceramics, γ-ray ^137^Cs, 0.75, 1, 2 μs time gate, respectively. (**b**) Comparison of relative LY values of Ce, Pr:LuAG crystals and ceramics at different shaping times, 0.75, 1, 2 μs, respctively.

We investigated the pulse height spectra of the as grown Ce, Pr:LuAG crystals and ceramics under γ ray excitation (662 keV, ^137^Cs source) at 0.75 μs, 1 μs, 2 μs, respectively Figure 8 a gives the comparison of full-energy peaks at the longest shaping time of 2 μs. It can be seen that the highest channel number reaches 283, which belongs to the Ce, Pr:LuAG ceramics with post 1450 °C–10 h air annealing. The air annealing process improved the overall scintillation efficiency at different time gates, which is inconsistent with our previous studies [5,25,32]. However, for the annealed Ce, Pr:LuAG crystals, no obvious full-energy peak can be observed, considering the emission of Ce^3+^ and Pr^3+^ existed in PL and XEL spectra, although their intensities degraded. Then the heavy light scattering effects introduced by both cracks and inclusions, as well as some possible atomic-level defects, might be the possible cause of the absence of scintillation LY.

The emission peak of Ce,Pr:LuAG in this work is comparable to that of Ce:GGAG, which is centered at around 550 nm with a wide emission band [2,28]. To calculate the relative LY of those Ce, Pr:LuAG samples, we compared their corresponding channel numbers of the pulse height peaks [28] to a standard Ce:GGAG crystal (LY 58,000 pho/MeV, size: 27 × 15 × 2.37 mm^3^) with a peak at channel number 481.6, FWHM 33.60, and E.R 6.98%. Figure 8b shows the comparisons of the calculated LY values of Ce, Pr:LuAG crystals, and ceramics at different shaping times. The relative LY values increased with increasing shaping time. The maximum of relative LY reaches 28,422 pho/MeV at 2 μs time gate in as grown crystal while 34,112 pho/MeV at 2 μs in ceramic. The LY values of Ce:LuAG crystals are previously reported to be 14,000–26,000 pho/MeV [33,34]. We also previously reported the scintillation performance of Ce:LuAG transparent ceramics fabricated by vacuum sintering method [5,35] or translucent Ce:LuAG ceramics by spark plasma sintering (SPS) method [26]; their LY values ranged from 6000–10,000 pho/MeV or 4.36 folds of BGO crystal. Besides, the highest LY value reported in Pr:LuAG crystal was 33,000 pho/MeV at 3 μs [4] or Pr:LuAG ceramic 28,500 pho/MeV at 3 μs [36], both of which were optimized by using Y co-doping. Those above LY values are still far from the theoretical LY of LuAG, i.e., 60,000 pho/MeV based on the Bartram−Lempicki theory [9,10]. Comparatively, Ce^3+^ and Pr^3+^ co-doped LuAG in this work thus have an enhanced scintillation efficiency which might be ascribed to the energy transitions between Ce^3+^ and Pr^3+^. Furthermore, the relative LY value (LY2/LY0.75) between the longest 2 μs and shortest 0.75 μs time gate also reveals the extent of the slow scintillation decay component of the samples [26]. As shown in Table 2, they are 134%, 137%, and 147% for as sintered Ce, Pr:LuAG annealed ceramic, as sintered ceramic, and as grown crystal, respectively. The results demonstrated a comparatively lower slow decay component regarding the fast Ce, Mg:LuAG ceramics (50% difference from 0.5 μs to 10 μs) [35]. The co-doping of Pr to Ce:LuAG thus provides a potential strategy to optimize the scintillation decay performance of LuAG-based scintillators.

## 4. Conclusions

Ce^3+^ and Pr^3+^ co-doped LuAG scintillator crystals and ceramics with a nominal concentration of 0.5 at.% Ce and 0.1 at.% Pr were prepared using the OFZ method and solid-state reactive vacuum sintering technology, respectively, with the same raw materials and powder process conditions. A comparative study found that both technologies could generate a pure cubic garnet phase. As evidenced by the absorbance and emission spectra, both Ce^3+^ and Pr^3+^ co-existed in the crystals and ceramics. The in-line transmittance of Ce, Pr:LuAG ceramics in the visible light range is relatively low, at around 20%, when measured at a thickness of 1.2 mm, which was attributed to the lack of sintering aids. The transmittance of crystal grown by the OFZ method was better at 65%, 2 mm thickness, and further optimization can be expected through modification of crystal growth parameters.

Although crystals were more transparent than ceramics, the latter showed superior scintillation light yield and decay. The highest LY of Ce, Pr:LuAG ceramics reached 34,112 pho/MeV at 2 μs time gate. Ce, Pr co-doped LuAG also proved to have a comparable scintillation decay with that of previously reported fast Ce, Mg co-doped LuAG ceramics.

The superiority of ceramics can be attributed to the lower real Ce^3+^ and Pr^3+^ concentrations in crystals regarding nominal concentration due to the segregation coefficient effect. Ce,Pr: LuAG ceramics were proved to keep the nominal concentration well, as determined by the ICP-OES results.

Consequently, the co-doping of Ce and Pr in LuAG is a promising prospect for a fast scintillator, and future studies should be conducted to explore the influence of Pr^3+^ concentration.

## Figures and Tables

**Table 1:** The experimental activator concentrations of the as grown Ce, Pr:LuAG crystals and ceramics by ICP-OES measurement.

		As Sintered Ceramic	As Grown Crystal	Crystal with 1000 °C-5 h-Air Annealing	Crystal with 1000 °C-10 h-Air Annealing
Experimental concentration by ICP-OES (wt.%)	Ce^3+^	0.26	0.13	0.058	0.056
	Pr^3+^	0.046	0.032	0.018	0.017

**Table 2:** Channel number and calculated LY values (pho/MeV) using a standard Ce:GGAG crystal (LY 58,000 pho/MeV, size: 27 × 15 × 2.37 mm^3^) with the corresponding peak at channel number 481.6, FWHM 33.60, and E.R 6.98% as reference.

	As Sintered Ceramic	Ceramic, 1450 °C-10 h-Air Annealed	As Grown Crystal
Channel number/Calculated Lyat 0.75 μs, pho/MeV	143/17,222	211/25,411	161/19,389
Channel number/Calculated LY at 1 μs, pho/MeV	162/19,510	240/28,904	186/22,400
Channel number/Calculated LY at 2 μs, pho/MeV	196/23,605	283/34,082	236/28,422
LY2/LY0.75	137%	134%	147%

## References

[1] Dujardin C., Auffray E., Bourret-Courchesne E., Dorenbos P., Lecoq P., Nikl M., Vasil’ev A.N., Yoshikawa A., Zhu R.Y. (2018). Needs, Trends, and Advances in Inorganic Scintillators. IEEE Trans. Nucl. Sci..

[2] Wu T., Wang L., He H., Wang H., Shen H., Liu Q., Shi Y. (2021). Research progress of Lu_3_Al_5_O_12_-based scintillation ceramics. Chin. J. Lumin..

[3] Nikl M., Yoshikawa A., Kamada K., Nejezchleb K., Stanek C.R., Mares J.A., Blazek K. (2013). Development of LuAG-based scintillator crystals—A review. Prog. Cryst. Growth Charact. Mater..

[4] Drozdowski W., Brylew K., Wojtowicz A.J., Kisielewski J., Świrkowicz M., Łukasiewicz T., de Haas J.T.M., Dorenbos P. (2014). 33000 photons per MeV from mixed (Lu_0.75_Y_0.25_)_3_Al_5_O_12_:Pr scintillator crystals. Opt. Mater. Express.

[5] Shi Y., Zhao Y., Cao M., Chen H., Hu Z., Chen X., Zhang Z., Liu Q. (2020). Dense Ce^3+^ doped Lu_3_A1_5_O_12_ ceramic scintillators with low sintering adds: Doping content effect, luminescence characterization and proton irradiation hardness. J. Lumin..

[6] Shi Y., Nikl M., Feng X., Mares J.A., Shen Y., Beitlerova A., Kucerkova R., Pan Y., Liu Q. (2011). Microstructure, optical, and scintillation characteristics of Pr^3+^ doped Lu_3_Al_5_O_12_ optical ceramics. J. Appl. Phys..

[7] Dai Y., Zhang Z., Wang X., Lu Z., Kou H., Su L., Wu A. (2021). Growth and Characterization of Ce-Doped LuAG Single Crystal Fibers from Transparent Ceramics by Laser-Heated Pedestal Method. Crystals.

[8] Zorenko T., Gorbenko V., Vozniak T., Heinrich S., Huber G., Zorenko Y. (2020). Comparison of the luminescent properties of LuAG:Ce films grown by pulse laser deposition and liquid phase epitaxy methods using synchrotron radiation excitation. Opt. Mater..

[9] Dorenbos P. (2010). Fundamental Limitations in the Performance of Ce^3+^-, Pr^3+^-, and Eu^2+^-Activated Scintillators. IEEE Trans. Nucl. Sci..

[10] Ogieglo J.M., Katelnikovas A., Zych A., Juestel T., Meijerink A., Ronda C.R. (2013). Luminescence and Luminescence Quenching in Gd_3_(Ga,Al)_5_O_12_ Scintillators Doped with Ce^3+^. J. Phys. Chem. A.

[11] Zhu R.-Y. (2019). Ultrafast and Radiation Hard Inorganic Scintillators for Future HEP Experiments. J. Phys. Conf. Ser..

[12] Hu C., Yang F., Zhang L., Zhu R.-Y., Kapustinsky J., Li X., Mocko M., Nelson R., Wender S., Wang Z. (2022). Hadron-Induced Radiation Damage in Fast Heavy Inorganic Scintillators. Instruments.

[13] Shen Y.Q., Shi Y., Pan Y.B., Feng X.Q., Wu L.X., Kou H.M., Zhang Z.M., Wei L. (2014). Fabrication and 2D-mapping of Pr: Lu_3_Al_5_O_12_ Scintillator Ceramics with High Light Yield and Fast Decay Time. J. Inorg. Mater..

[14] Nikl M., Ogino H., Krasnikov A., Beitlerova A., Yoshikawa A., Fukuda T. (2005). Photo- and radioluminescence of Pr-doped Lu_3_Al_5_O_12_ single crystal. Phys. Status Solidi.

[15] Yanagida T., Fujimoto Y., Kurosawa S., Kamada K., Takahashi H., Fukazawa Y., Nikl M., Chani V. (2013). Temperature Dependence of Scintillation Properties of Bright Oxide Scintillators for Well-Logging. Jpn. J. Appl. Phys..

[16] Ding Z., Ying S., Ling-Cong F., De-Bao L., Ze-Qing S., Jia-Yue X. (2016). Fabrication and Optical Properties of Ce, Pr Co-doped LuAG Transparent Ceramics. J. Inorg. Mater..

[17] Xue-jiao N., Jia-Yue X., Ding Z., Shu-Xian W., Huai-Jin Z. (2015). Synthesis and Growth of Ce, Pr: YLuAG Crystal for LED Application. J. Inorg. Mater..

[18] Herzog J.M., Witkowski D., Rothamer D.A. (2021). Combined scattering-referenced and co-doped aerosol phosphor thermometry using the Ce,Pr:LuAG phosphor. Appl. Phys. B-Lasers Opt..

[19] Herzog J.M., Witkowski D., Rothamer D.A. (2021). Characterization of the Ce, Pr:LuAG phosphor for Co-doped aerosol phosphor thermometry. J. Lumin..

[20] Herzog J.M., Witkowski D., Rothamer D.A. (2021). Combustion-relevant aerosol phosphor thermometry imaging using Ce, Pr:LuAG, Ce: GdPO_4_, and Ce: CSSO. Proc. Combust. Inst..

[21] Liu S., Feng X., Zhou Z., Nikl M., Shi Y., Pan Y. (2014). Effect of Mg^2+^ co-doping on the scintillation performance of LuAG: Ce ceramics. Phys. Status Solidi Rapid Res. Lett..

[22] Mares J.A., Nikl M., Beitlerova A., Horodysky P., Blazek K., Bartos K., D’Ambrosio C. (2012). Scintillation Properties of Ce^3+^- and Pr^3+^- doped LuAG YAG and mixed Lu_x_Y_1-x_AG garnet crystals. IEEE Trans. Nucl. Sci..

[23] Petrosyan A.G., Ovanesyan K.L., Sargsyan R.V., Shirinyan G.O., Abler D., Auffray E., Lecoq P., Dujardin C., Pedrini C. (2010). Bridgman growth and site occupation in LuAG: Ce scintillator crystals. J. Cryst. Growth.

[24] Pejchal J., Guguschev C., Schulze M., Jary V., Mihokova E., Rubesova K., Jakes V., Barta J., Nikl M. (2019). Luminescence and scintillation properties of strontium hafnate and strontium zirconate single crystals. Opt. Mater..

[25] Kurosawa S., Shishido T., Sugawara T., Nomura A., Yubuta K., Suzuki A., Murakami R., Pejchal J., Yokota Y., Kamada K. (2015). Scintillation properties of Ce:(La,Gd)_2_Si_2_O_7_ at high temperatures. Nucl. Instrum. Methods Phys. Res. Sect. A Accel. Spectrometers Detect. Assoc. Equip..

[26] Shi Y., Shichalin O., Xiong Y., Kosyanov D., Wu T., Zhang Q., Wang L., Zhou Z., Wang H., Fang J. (2022). Ce^3+^ doped Lu_3_Al_5_O_12_ ceramics prepared by spark plasma sintering technology using micrometre powders: Microstructure, luminescence, and scintillation properties. J. Eur. Ceram. Soc..

[27] Hu Z.W., Cao M.Q., Chen H.H., Shi Y., Kou H.M., Xie T.F., Wu L.X., Pan Y.B., Feng X.Q., Vedda A. (2017). The role of air annealing on the optical and scintillation properties of Mg co-doped Pr:LuAG transparent ceramics. Opt. Mater..

[28] Wu T., Wang L., Shi Y., Xu T.-Z., Wang H., Fang J.-H., Ni J.-Q., He H., Wang C.-Y., Wan B. (2022). Fast (Ce,Gd)_3_Ga_2_Al_3_O_12_ scintillators grown by the optical floating zone method. Cryst. Growth Des..

[29] Blasse G., Grabmaier B.C., Blasse G., Grabmaier B.C. (1994). Luminescent Materials.

[30] Nikl M., Mares J.A., Solovieva N., Li H.L., Liu X.J., Huang L.P., Fontana I., Fasoli M., Vedda A., D’Ambrosio C. (2007). Scintillation characteristics of Lu_3_A_l5_O_12_: Ce optical ceramics. J. Appl. Phys..

[31] Nikl M., Laguta V.V., Vedda A. (2008). Complex oxide scintillators: Material defects and scintillation performance. Phys. Stat. Sol..

[32] Chen X., Hu Z., Dai J., Chen H., Shi Y., Kou H., Wang T., Vedda A., Beitlerova A., Kucerkova R. (2018). The influence of air annealing on the microstructure and scintillation properties of Ce, Mg: LuAG ceramics. J. Am. Ceram. Soc..

[33] Nikl M., Kamada K., Babin V., Pejchal J., Pilarova K., Mihokova E., Beitlerova A., Bartosiewicz K., Kurosawa S., Yoshikawa A. (2014). Defect Engineering in Ce-Doped Aluminum Garnet Single Crystal Scintillators. Cryst. Growth Des..

[34] Chen X., Hu Z., Feng Y., Liu X., Chen H., Shi Y., Kucerkova R., Beitlerova A., Nikl M., Li J. (2019). Luminescence and scintillation characteristics of cerium doped Gd_2_YGa_3_Al_2_O_12_ ceramics. Opt. Mater..

[35] Liu S., Mares J.A., Babin V., Hu C., Kou H., D’Ambrosio C., Li J., Pan Y., Nikl M. (2017). Composition and properties tailoring in Mg^2+^ codoped non-stoichiometric LuAG:Ce,Mg scintillation ceramics. J. Eur. Ceram. Soc..

[36] Hu C., Feng X., Li J., Ge L., Zhang Y., Kou H., Xu J., Pan Y. (2017). Fabrication, optical and scintillation properties of (Lu_0.75_,Y_0.25_ ) AG:Pr ceramic scintillators. Opt. Mater..

